# The Study of a Validated Assessment Scale for a Localized Submental Fat Volume

**DOI:** 10.3390/jcm12031226

**Published:** 2023-02-03

**Authors:** Joon-Seok Oh, Jinil Choi, Jong-Woo Choi, Dae-Hyun Lew, Tai-Suk Roh, Seung-Yong Song, Young-Seok Kim, Hojin Park, Sang-Woong Youn, Shinhyuk Kang, Jihyun Park, Jeongseok Oh, Chan-Yeong Heo

**Affiliations:** 1 Department of Plastic and Reconstructive Surgery, Seoul National University Hospital, Seoul National University College of Medicine, Seoul 03080, Republic of Korea; 2 Department of Plastic and Reconstructive Surgery, Asan Medical Center, University of Ulsan College of Medicine, Seoul 05505, Republic of Korea; 3 Department of Plastic and Reconstructive Surgery, Department of Medical Education, Yonsei University College of Medicine, Seoul 03722, Republic of Korea; 4 Department of Plastic Surgery, Institute for Human Tissue Regeneration, Yonsei University College of Medicine, Seoul 06273, Republic of Korea; 5 Department of Plastic and Reconstructive Surgery, Gangnam Severance Hospital, Yonsei University College of Medicine, Seoul 06273, Republic of Korea; 6 Department of Dermatology, Seoul National University Bundang Hospital, Seoul National University College of Medicine, Seoul 03080, Republic of Korea; 7 Department of Plastic and Reconstructive Surgery, Chung-Ang University Hospital, Chung-Ang University College of Medicine, Seoul 06973, Republic of Korea; 8 Research and Development Team, AMI Pharm Co., Ltd., Seongnam 13487, Republic of Korea; 9 Department of Plastic and Reconstructive Surgery, Seoul National University Bundang Hospital, Seoul National University College of Medicine, Seongnam 13620, Republic of Korea

**Keywords:** submental fat, rating scale, aging face

## Abstract

Background: This aim of this study was to develop an objective tool for rating submental fat applied to Koreans. Methods: The study was conducted between April 2019 and October 2019. A total of 92 subjects were enrolled in the study. Clinical photos of the subjects were categorized using validated CR-SMFRS by three plastic surgeons and one dermatologist. The categorized photos were then shown to six different plastic surgeons for evaluation. Results: The Cohen’s kappa value for the six raters were 0.830, 0.742, 0.703, 0.907, 0.862, and 0.793 with statistical significance (*p* < 0.001). ICC value was between 0.860 and 0.966 (*p* < 0.001). Since the Cohen’s value and ICC were above 0.6 for all raters, the ratings performed by all six raters were used in the analysis. The ICC values between raters were between 0.899 and 0.902. Conclusions: We came up with a set of reference photos that can be used for submental fat rating scale applicable to Korean subjects. Level of Evidence: II.

## 1. Introduction

In an era of global population aging, people are becoming more interested in rejuvenating their facial cosmetic appearance [1]. Submental fat, also called a double chin, is one of the most significant aspects of this phenomenon. Protrusion and/or accumulation of submental fat is a common cause of less attractive attributes [2]. It may present as skin sagging and unattractive facial contour [3]. The pathophysiology is a combination of natural aging processes, lifestyle, and/or genetic factors [2]. Typical remedies to improve submental fat contour include exercise and diet control. However, many patients require non-surgical means of contour improvement. Hence, minimally invasive procedures such as botulinum and filler injections, lasers, and peeling applications are on a dramatic rise along with surgical procedures [4]. Current treatment methods rely on non-surgical methods using medical devices, various injections (often off-label), and surgical liposuction. Currently, patients request more effective but less risky measures over surgical treatments or off-label injections. In 2015, the US FDA approved Kybella (ingredient name: desoxycholic acid, clinical trial code name: ATX-101), developed by Kythera biopharmaceuticals INC, to reduce submental fat [5]. As the validated submental fat rating system of this ATX-101 clinical trial was customized for Caucasians, we believe it does not suffice for Far East Asians [5]. Asian skin dermis is thicker and contains greater collagen than Caucasian skin and has less laxity problem. Since Asian skin contains darker pigment, they are better protected against photoaging. However, Asians have weaker skeletal support, bulkier soft tissue, and more malar fat, making Asian skin more vulnerable to gravitational force [6]. Efforts to establish an objective evaluation scale for Asians are increasing to meet the corresponding demand for facial rejuvenation [7].

The purpose of this study was to develop and validate an objective submental fat rating tool for Koreans. For this purpose, the Clinician-Reported Submental Fat Rating Scale (CR-SMFRS) used for the trial of ATX-101 was used as a reference to evaluate the inter-rater and intra-rater reliability of a submental fat rating scale conducted on Korean subjects.

## 2. Materials and Methods

The study was conducted in accordance with the Declaration of Helsinki and Korean Good Clinical Practice (KGCP). All aspects of the study were approved by the Institutional Review Board (IRB No. B-1901/514-303). Prior to their participation, subjects were informed of the purpose and potential risks associated with the trial through a consent form approved by the IRB. The consent form was signed, and a copy was given to the subject accordingly.

The study was conducted between 1 April 2019 and 18 October 2019. A total of 92 subjects were enrolled in this study. Subjects included any male or female patients between the ages of 15 and 65 years who agreed to sign the consent form. The exclusion criteria are listed in Table 1.

The tools used for the trial of ATX1-1, such as the Clinician-Reported Submental Fat Rating Scale (CR-SMFRS), Patient-Reported Submental Fat Rating Scale (PR-SMFRS), Subject-Self Rating Scale (SSRS), and Patient-Reported Submental Fat Impact Scale (PR-SMFIS), were translated with linguistic validation and renamed for the study. The renamed tools are presented in Table 2.

**Figure 1:**
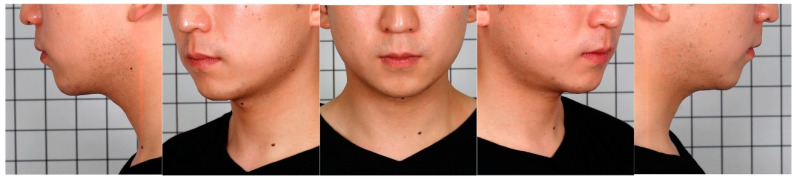
Sample of a set of two-dimensional clinical photos of a male patient.

**Figure 2:**
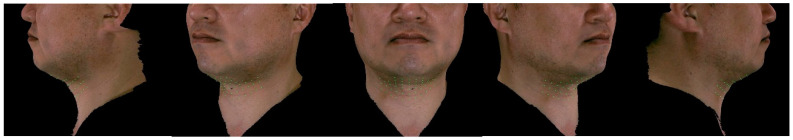
Sample of a set of three-dimensional clinical photos of a male patient.

**Figure 3:**
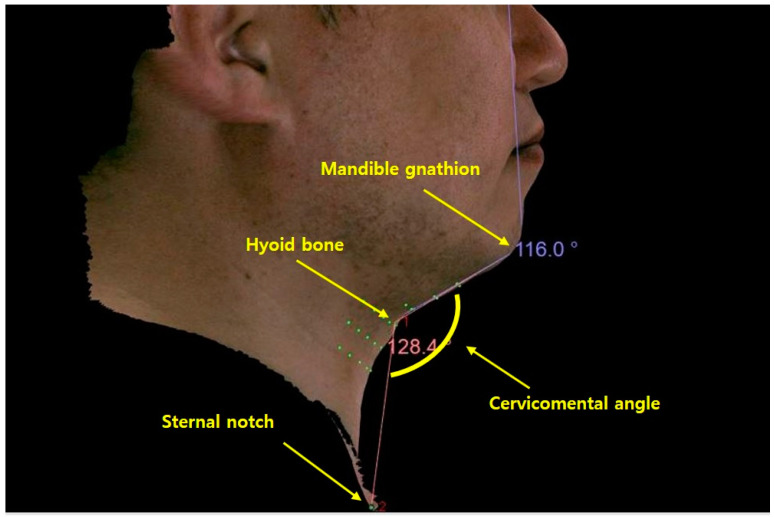
Measuring cervicomental angle using three-dimensional analysis.

On the first day of the visit, the subjects signed the consent form. Height and body weight measurements of the subjects were also taken. Two photographers took two- and three-dimensional photographs of all the enrolled subjects. Five two-dimensional photos were taken in front, left/right lateral, and left/right oblique views, as shown in Figure 1. Photographs were taken using a Canon EOS1 DX MARK2 camera. Six photos were taken in front, left/right lateral, left/right oblique, and worm’s eye view for three-dimensional analysis, as shown in Figure 2. Morpheus Plastic Solution (version 3.0) software and a structured light scanner for the Morpheus 3D scanner were used for three-dimensional analysis. The cervicomental angle—the angle formed by a straight line drawn from the hyoid bone to the mandibular gnathion point and a second straight line drawn from the hyoid bone to the sternal notch—was measured using a three-dimensional analysis, as shown in Figure 3. This is crucial for defining the lateral shape of the chin and neck [8]. The ideal angle was 105–120° [9,10].

The subjects completed the SSSS, SR-SMFPIS, DAS 24, BIQLI, blind SR-SMFRS, and unblind SR-SMFRS. If the clinical photograph taken was considered inappropriate for evaluation, the subject was asked to revisit for a new set of clinical photographs. The clinical photos taken were used to evaluate the subjects’ submental fat using the ATX-101 validated SMFRS used as a reference. Thus, clinical photos were taken using standardized methods from two photographers.

The research team was composed of three plastic surgeons and one dermatologist from one institution. The evaluation team was composed of six plastic surgeons from three different institutions. The photos, without any patient information, were sent to the research team of three plastic surgeons and one dermatologist. After being familiarized with the validated CR-SMFRS used in the ATX-101 trial, the research team categorized the set of photos into the different grades used in CR-SMFRS. Categorization was performed so that at least 10 subjects were included in each grade. This set of photos was then provided to the rating team.

The rating team, composed of six plastic surgeons, was informed about the validated CR-SMFRS used in the ATX-101 trial. The blind raters were given clinical photos of subjects, graded by the research team, in a random arrangement and were instructed to stratify them based on the validated CR-SMFRS guideline. Four weeks later, the rater team, which was still blinded, evaluated the same set of photos that were rearranged randomly. The rating scale utilized by the rater team was ER-SMFRS. The intra-rater reliability was analyzed using Cohen’s kappa coefficient, and any rater with a kappa value < 0.6 was excluded. Inter-rater reliability was analyzed using intraclass correlation coefficient (ICC). Data derived from a rater with values < 0.6 were excluded from the analysis.

All statistical analyses were performed using R software (version 4.0). For continuous variables, the mean, standard deviation, and minimum and maximum values were calculated. For categorical variables, the frequencies and percentages were calculated. Data with missing values were excluded from the analysis. All test statistics used in the analysis of this data were the results of a two-sided test, and the statistical confidence level was based on a multiplicity correction of 0.05.

The correlation of unblind SR-SMFRS with blind SR-SMFR; ER-SMFRS with blind SR-SMFRS, unblind SR-SMFRS, SSSS, SR-SMFPIS, DAS24, BIQLI, and cervicomental angle; blind SR-SMFR with SSSS, SR-SMFPIS, DAS 24, BIQLI, and cervicomental angle; unblind SR-SMFR with SSS, SR-SMFPIS, DAS 24, BIQLI, and cervicomental angle; and SSSS with SR-SMFPIS, DAS 24, BIQLI, and cervicomental angle was evaluated.

## 3. Result

**Figure 4:**
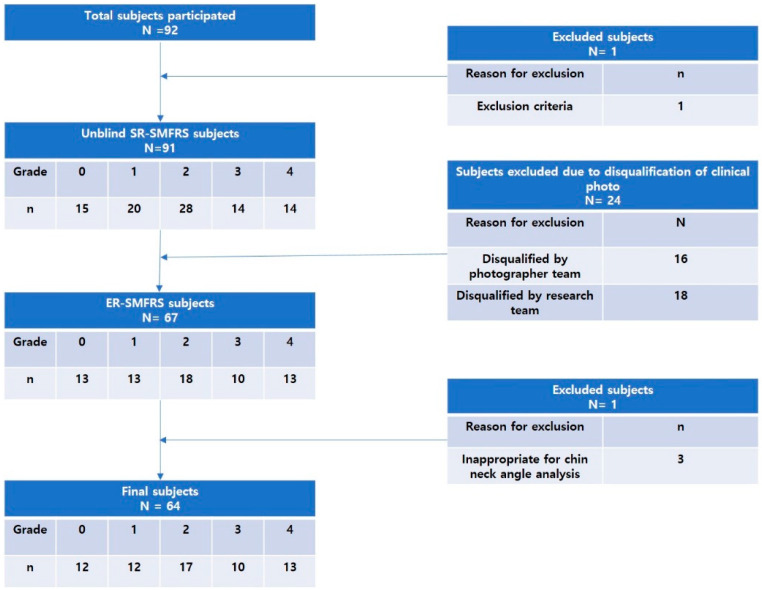
Flowchart of subjects enrolled in the trial.

Initially, 92 patients agreed to participate and were enrolled in the study. Among them, one was excluded due to a violation of selection/exclusion criterion (the subject’s age was above 65 years). Based on the unblind SR-SMFRS, 15 subjects were classified as grade 0, 20 as grade 1, 28 as grade 2, 14 as grade 3, and 14 as grade 4, respectively. The photography team excluded 16 subjects due to quality issues of photos including poor subject position, poor image quality, inadequate Frankfort horizontal plane, and insufficient light exposure. The research team excluded eight subjects: three due to low inter-rater match, two due to acne scar, one due to lax chin skin, one due to short chin, and one due to mandibular prognathism. Three additional subjects were excluded due to inappropriate cervicomental angle in the three-dimensional analysis. The hyoid bone and sternal notch were marked on the subjects before 3D scanning. However, the markers were inadvertently deleted during the scanning process for the three subjects excluded from the cervicomental angle analysis. The exclusion process is shown in Figure 4. Finally, a photobook of 64 patients was provided to the rater team. Based on the evaluated ER-SMFRS, 0 subjects were grade 0, 19 were grade 1, 7 were grade 2, 15 were grade 3, and 13 were grade 4.

The Cohen’s kappa values for the six raters were 0.830, 0.742, 0.703, 0.907, 0.862, and 0.793, respectively (*p* < 0.001). The ICC values were between 0.860 and 0.966 (*p* < 0.001). Since the Cohen’s values and ICC were above 0.6, for all raters, the ratings performed by all six raters were used in the analysis. The ICC values between the raters were between 0.899 and 0.902.

The gradings performed by the rater team, corresponding to ER- SMFRS, were 0.11 ± 0.12 for grade 0, 1.01 ± 0.27 for grade 1, 2.06 ± 0.26 for grade 2, 3.01 ± 0.31 for grade 3, and 3.75 ± 0.23 for grade 4. The difference between grades was statistically significant (*p* < 0.001). Age, BMI, and sex information are outlined in Table 3. Age was highest in grade 2, and BMI was highest in grade 4. Age and BMI both increased with increasing grade (*p* = 0.001, *p* < 0.001, respectively). The ratio of female subjects was highest in grade 0. The ratio of male subjects was highest in grade 4, and the ratio increased with an increase in the grade (*p* < 0.003).

The correlations of among ER-SMFRS with blind SR_SMFRS, unblind SR-SMFRS, SSSS, SR-SMFPIS, DAS24, BIQLI, and cervicomental angle are outlined in Table 4, Table 5, Table 6, Table 7, Table 8, Table 9 and Table 10.

## 4. Discussion

Several studies have reported scales to evaluate a person’s facial appearance due to aging. Shoshani et al. built a scale to evaluate nasolabial wrinkles [11]. The scale is a reliable method for assessing nasolabial skin folds with good intra-and inter-rater reliability. Carruthers et al. developed a validated grading scale for brow positioning, forehead lines, lip fullness, marionette lines, crow’s feet, upper face, lower face, neck volume, and midface [12]. In 1 study, a composite scale was developed to assess the whole global face, with 12 raters evaluating 50 subjects using clinical photos [7]. The scale was reliable, with high inter-and intra-rater reliability. Actual age was highly correlated with the scale. The raters ranked the lower face unit, consisting of the jaw line, marionette lines, nasolabial folds, and oral commissures as the most problematic facial aging part. This part of the face showed the highest correlation with subject age.

Thus, it is observed that important factors of facial aging are mainly present in the lower part of the face. Although the scale did not specifically include submental fat, it is implied that the lower part of the face is a crucial part of facial rejuvenation.

The current validated evaluation method for submental fat is the one used to evaluate submental fat in Westerners in the ATX-101 clinical trial performed by Kythera biopharmaceuticals INC under FDA approval [5]. It is classified into five stages from “None” to “Extreme” according to the amount of submental fat. The facial bones are anatomically different between Caucasians and East Asians [13]. Therefore, an objective evaluation scale was developed according to the amount of submental fat in Asians, especially Koreans, to obtain credibility in an evaluation tool that can be used in clinical research involving submental fat treatment.

By comparing the various demographic information that may affect submental fat among the subjects, it was confirmed that there were meaningful differences in submental fat depending on the subject’s age, BMI, and sex. Age was highest in subjects categorized as grade 2. BMI was highest in grade 4, and both age and BMI increased with increasing submental fat grade (*p* = 0.001, *p* < 0.001). Regarding sex, the ratio of women was the highest in grade 0, and the ratio of men was the largest in grade 4. Based on the results, it was found that when the submental fat grade increased, the ratio of men was high. Therefore, when evaluating the degree of submental fat, it is necessary to consider the factors of age, sex, and BMI.

The rater team, independent of the research team, was composed of six plastic surgeons at three different institutions. The team was trained and informed about the validated CR-SMFRS used in the ATX-101 trial, and the team evaluated the front, left/right lateral, and left/right oblique view clinical photos of 64 subjects using ER-SMFRS. The intra-rater reliability within the six raters ranged from 0.703 to 0.907, which were in “excellent agreement” and “perfect agreement.” The Cohen’s kappa values for each rater were 0.830, 0.742, 0.703, 0.907, 0.862, and 0.793, respectively, with statistical significance (*p* < 0.001). No evaluators were excluded because there were no raters with Cohen’s kappa values below 0.6. The ICC of the results of submental fat rating among raters also ranged from 0.899 to 0.902 with a 95% confidence interval, showing almost perfect agreement. Both the 1st and 2nd evaluations conducted 4 weeks apart were found to have high reliability and reproducibility: 0.11 ± 0.12 for grade 0, 1.01 ± 0.27 for grade 1, 2.06 ± 0.26 for grade 2, 3.01 ± 0.31 for grade 3, and 3.75 ± 0.23 for grade 4. The difference between grades was statistically significant (*p* < 0.001). Clinicians in the esthetic field who have been trained with this highly reliable evaluation method are expected to be able to objectively evaluate the degree of improvement of submental fat during clinical studies and procedures.

**Figure 5:**
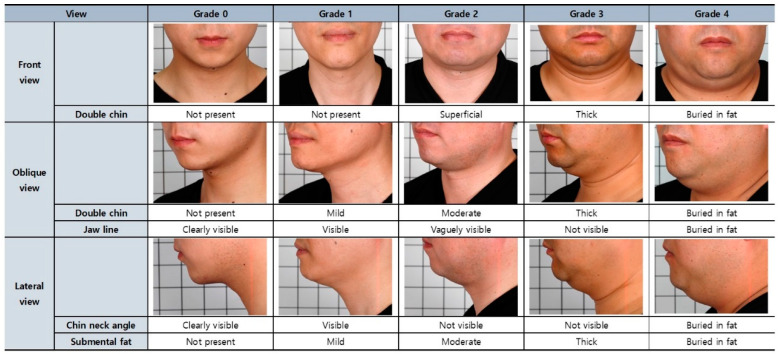
Reference for evaluating submental fat of male subjects.

**Figure 6:**
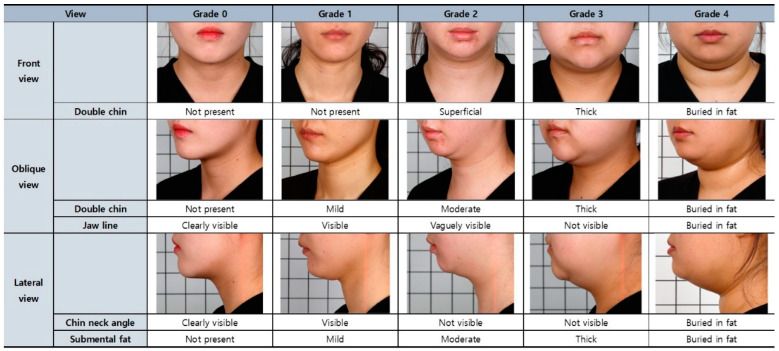
Reference for evaluating submental fat of female subjects.

In this study, all 64 subjects evaluated through quality control, screening, and validation processes have secured high reliability and can be applied as reference photos that can be used for the submental fat rating scale. Among the 64 subjects, the subjects were selected in the order of the highest degree of agreement among the raters for each grade scale, and their clinical photos were expected to be used as reference for the Korean Submental Fat Rating Scale. An example of a set of clinical photos corresponding to each grading scale is shown in Figure 5 for male subjects and Figure 6 for female subjects.

The correlation coefficient between blind SR-SMFRS, which was performed while maintaining the blindness of subjects by not providing information on validated CR-SMFRS used in the ATX-101 trial, and unblind SR = SMFRS, which was performed without blinding the subjects by providing a photobook of validated CR-SMFRS used in the ATX-101 clinical trial, was 0.859 with statistical significance (*p* < 0.001). This implied that the subject could objectively self-evaluate the submental fat with a certain precision.

The correlation coefficient between the ER-SMFRS and blind SR-SMFRS was 0.689 (*p* < 0.001). The correlation coefficient between the ER-SMFRS and unblind SR-SMFRS was 0.757 (*p* < 0.001). Although ER-SMFRS correlated with both blind and unblind SR-SMFRS with statistical significance, the correlation was stronger with unblind SR-SMFRS. Thus, if validated CR-SMFRS were provided to subjects for reference, self-evaluation of submental fat would be more objective.

The correlation between submental fat grading and cervicomental angle, a representative index of esthetic submental fat assessment, was evaluated using three-dimensional photo analysis. The correlation coefficients between the cervicomental angle and ER-SMFRS, blind SR-SMFRS, and unblind SR-SMFRS were 0.694 (*p* < 0.01), 0.426 (*p* < 0.001), and 00.497 (*p* < 0.001), respectively. The average cervicomental angles were 113 ± 5.12° for ER-SMFRS grade 0 subjects and 139.8 ± 13.22° for ER-SMFRS grade 4 subjects. The difference between grade 0 and 4 subjects varied from 8 to 44°. Due to the variability in the anatomical shape of each subject, there was a limitation in arriving at a reliable, objective cervicomental angle range for each submental fat grade.

There are several limitations to the study. First, all evaluations were made using clinical photos only. Thus, skin laxity was evaluated only by photo. The study would have been much more precise if patients were evaluated in person. The study excluded any patient older than 65 years old. As submental laxity occurs more frequently in aged patients, a study including patients older than 65 years old would be a more comprehensive tool that can be used for patients in a wider range of age. Furthermore, the scale may not be generalizable to the entire East Asian population since the study included only Korean patients. At last, this study excluded patients who received any type of non-surgical or surgical treatment of submental fat. Thus, it may not be accurate in evaluating the effectiveness of any rejuvenation therapy to reduce submental fat.

## 5. Conclusions

Based on the results derived from this study, it is possible to produce an evaluation guideline that includes reference photos and criteria for a reliable submental fat rating scale applicable to subjects, especially Koreans, to evaluate the improvement of submental fat. Furthermore, a survey that evaluates the quality of life associated with submental fat can also be developed. This validated submental fat rating scale can be applied as a tool for evaluating the effectiveness of clinical trials for the improvement of submental fat. It can be utilized as a diagnostic index that can be applied in the field of cosmetic plastic surgery to provide customized yet reliable information on submental fat to both patients and medical staff.

## Figures and Tables

**Table 1:** Exclusion criteria.

Exclusion Criteria
Has beard in the submental area History of surgery around the chin and neck area (e.g., two jaw operation, liposuction) History of any injection (Botox, filler, Polyen Phodphatidylcholine, mesotherapy, carboxy, laser) around the chin and neck area within 1 year Scar or previous operation wound around the chin and neck area Has HIV-associated lipodystrophy Lymphadenopathy or any other disease around chin and neck that may affect the evaluation Refused to take clinical photo Considered inappropriate for the study by examiner

**Table 2:** Scale used in the study.

Scale Name	Description
Blind Subject-Rated Submental-Rated Submental Fat Rating Scale (Blind SR-SMFRS) *	Subject’s self-evaluation of the submental fat independently without any information about Unbind SR-SMFRS or ER-SMFRS. Grade 0: No submental fat Grade 1: Slight amount of submental fat Grade 2: Moderate amount of submental fat Grade 3: Large amount of submental fat Grade 4: Very large amount of submental fat
Unblind Subject-Rated Submental Fat Rating Scale (Unblind SR-SMFRS) *	Subject’s self-evaluation of the submental fat with reference to validated CR-SMFRS from ATX-101 trial Grade 0: No submental fat Grade 1: Slight amount of submental fat Grade 2: Moderate amount of submental fat Grade 3: Large amount of submental fat Grade 4: Very large amount of submental fat
Evaluator-Rated Submental Fat Rating Scale (ER-SMFRS)	Examiner’s evaluation of subject’s submental fat with reference to validated CR-SMFRS from ATX-101 trial. The examiners are composed of 6 plastic surgeons.
Subject Self Satisfaction Scale (SSSS) *	Subject’s own satisfaction scale regarding submental fat Very dissatisfied (0), Dissatisfied (1), Slightly dissatisfied (2), Neutral (3), Slightly satisfied (4), Satisfied (5), Very satisfied (6)
Subject-Rated Submental Fat Psychological Impact Scale (SR-SMFPIS) *	Subject’s own evaluation of the psychological effect of the subject’s appearance of submental fat on the subject’s quality of life. It has been used in the clinical trial for ATX-101. Contains 6 items. Each item is composed of 10 points. For question 1, a higher point is a positive self-evaluation, while the remaining 5 questions are negative evaluation with higher points. Thus, for analysis, a reverse correlation was used to analyze for question 1. 1. How satisfied are you with your submental fat? 2. How much attention do you pay to your submental fat? 3. How much are you conscious about other people’s view on your submental fat? 4. How embarrassed are you about your submental fat? 5. How much older do you look due to submental fat? 6. How much overweight do you look due to submental fat?
Derriford Appearance Scale 24 (DAS 24)	Korean version of psychological measure of appearance concern validated for use in research and clinical settings. Contains 24 items. This survey originally takes into account the general physical appearance, but subjects were asked only to take into account their chin appearance. Each item is scored between −3 and +3.
Body Image Quality Life Inventory (BIQLI)	Self-evaluation of whether one’s own physical appearance gives negative/positive effect on one’s life. Very negative (−3), Moderately negative (−2), Mildly negative (−1), No effect (0), Mildly positive (+1), Moderately positive (+2), Very positive (3+). This survey originally takes into account the general physical appearance, but subjects were asked only to take into account their chin appearance.
Cervicomental angle	The angle formed by a straight line drawn from hyoid bone to mandible gnathion point and a second straight line drawn from hyoid bone to sternal notch.

* While maintaining the same position as when clinical photos were taken, the subject self-evaluated by looking in a mirror without distortion in the Frankfort horizontal plane, which was maintained in the horizontal position.

**Table 3:** Patient factor with respect to ER-SMFRS.

ER-SMFRS	Grade 0	Grade 1	Grade 2	Grade 3	Grade 4	Total	*p*-Value
Age Mean ± SD	30.3 ± 4.6	33.9 ± 8.4	44.4 ± 9.4	39.7 ± 8.8	40.9 ± 8.4	37.3 ± 9.2	0.001
BMI Mean ± SD	21.5 ± 2.5	22.9 ± 2.7	26.4 ± 4.7	31.4 ± 5.0	33.8 ± 4.2	27.1 ± 6.1	<0.001
Gender Number of Subjects (%)	10 (100.0)	19 (100.0)	7 (100.0)	15 (100.0)	13 (100.0)	64 (100.0)	
-Male	1 (10)	4 (21.0)	4 (57.1)	8 (53.3)	10 (76.9)	27 (42.2)	<0.003
-Female	9 (90)	15 (78.9)	3 (42.9)	7 (46.7)	3 (23.1)	37 (57.8)	

**Table 4:** Correlation of ER-SMFRS with blind SR_SMFRS, unblind SR-SMFRS, SSSS, SR-SMFPIS, DAS24, BIQLI, and cervicomental angle.

	Grade 0	Grade 1	Grade 2	Grade 3	Grade 4	Spearman Correlation (*p*-Value)
Blind SR-SMFRS	1.20 ± 1.13	1.26 ± 0.87	2.43 ± 0.98	2.80 ± 0.68	3.31 ± 0.75	0.689 (<0.001)
Unblind SR-SMFRS	0.60 ± 0.70	1.00 ± 0.82	2.14 ± 0.90	2.93 ± 1.10	3.38 ± 0.77	0.757 (<0.001)
SSSS	3.10 ± 1.45	3.47 ± 1.50	2.29 ± 1.25	1.87 ± 1.06	1.77 ± 1.42	−0.442 (<0.001)
SR-SMFPIS	27.80 ± 13.26	23.89 ± 13.75	34.00 ± 16.27	39.47 ± 12.68	39.62 ± 9.12	0.393 (0.001)
DAS24	43.80 ± 9.19	38.89 ± 6.61	43.86 ± 14.24	55.93 ± 14.79	54.69 ± 17.24	0.371 (0.003)
BIQLI	11.90 ± 20.28	19.32 ± 14.60	4.00 ± 19.97	1.20 ± 26.58	−9.23 ± 23.55	−0.371 (0.003)
Cervicomental angle	113.31 ± 5.15	117.88 ± 11.55	132.06 ± 12.16	132.50 ± 7.99	139.82 ± 13.22	0.694 (<0.001)

**Table 5:** Correlation of blind SR-SMFRS with unblind SR-SMFRS, SSSS, SR-SMFPIS, DAS24, BIQLI, and cervicomental angle.

		Unblind SR-SMFRS	SSSS	SR-SMFPIS	DAS24	BIQLI	Cervicomental Angle
Blind SR-SMFRS	Spearman correlation (Rho)	0.859	−0.654	0.742	0.507	−0.539	0.426
	*p*-value	<0.001	<0.001	<0.001	<0.001	<0.001	<0.001

**Table 6:** Correlation of unblind SR-SMFRS with SSSS, SR-SMFPIS, DAS24, BIQLI, and cervicomental angle.

		SSSS	SR-SMFPIS	DAS24	BIQLI	Cervicomental Angle
Unblind SR-SMFRS	Spearman correlation (Rho)	−0.570	0.620	0.534	−0.489	0.497
	*p*-value	<0.001	<0.001	<0.001	<0.001	<0.001

**Table 7:** Correlation of SSSS with SR-SMFPIS, DAS24, BIQLI, and cervicomental angle.

		SR-SMFPIS	DAS24	BIQLI	Cervicomental Angle
SSSS	Spearman correlation (Rho)	−0.674	−0.586	0.539	−0.176
	*p*-value	<0.001	<0.001	<0.001	0.165

**Table 8:** Correlation of SR-SMFPIS with DAS24, BIQLI, and cervicomental angle.

		ER-SMFRS	Blind SR-SMFRS	Unblind SR-SMFRS	SSSS
1	Spearman correlation (Rho)	0.413	0.710	0.584	−0.666
	*p*-value	<0.001	<0.001	<0.001	<0.001
2	Spearman correlation (Rho)	0.280	0.620	0.501	−0.569
	*p*-value	0.025	<0.001	<0.001	<0.001
3	Spearman correlation (Rho)	0.112	0.424	0.292	−0.373
	*p*-value	0.379	<0.001	0.019	0.002
4	Spearman correlation (Rho)	0.294	0.622	0.520	−0.565
	*p*-value	0.018	<0.001	<0.001	<0.001
5	Spearman correlation (Rho)	0.389	0.653	0.584	−0.472
	*p*-value	0.001	<0.001	<0.001	<0.001
6	Spearman correlation (Rho)	0.535	0.793	0.717	−0.662
	*p*-value	<0.001	<0.001	<0.001	<0.001

**Table 9:** Correlation of DAS24 with ER-SMFRS, blind SR-SMFRS, unblind SR-SMFRS, and SSSS.

		ER-SMFRS	Blind SR-SMFRS	Unblind SR-SMFRS	SSSS
A	Spearman correlation (Rho)	0.340	0.432	0.418	−0.524
	*p*-value	0.006	<0.001	<0.001	<0.001
B	Spearman correlation (Rho)	0.289	0.300	0.368	−0.534
	*p*-value	0.020	0.016	0.003	<0.001
C	Spearman correlation (Rho)	0.226	0.421	0.438	−0.462
	*p*-value	0.073	<0.001	<0.001	<0.001
D	Spearman correlation (Rho)	0.365	0.487	0.455	−0.533
	*p*-value	0.003	<0.001	<0.001	<0.001
E	Spearman correlation (Rho)	0.283	0.395	0.395	−0.417
	*p*-value	0.024	0.001	0.001	<0.001
F	Spearman correlation (Rho)	0.408	0.543	0.470	−0.605
	*p*-value	<0.001	<0.001	<0.001	<0.001
G	Spearman correlation (Rho)	0.441	0.505	0.492	−0.464
	*p*-value	<0.001	<0.001	<0.001	<0.001
H	Spearman correlation (Rho)	0.233	0.121	0.188	−0.315
	*p*-value	0.064	0.342	0.138	0.011
I	Spearman correlation (Rho)	−0.052	0.123	0.159	−0.217
	*p*-value	0.682	0.333	0.211	0.085
J	Spearman correlation (Rho)	0.229	0.222	0.198	−0.122
	*p*-value	0.068	0.078	0.117	0.338
K	Spearman correlation (Rho)	−0.019	0.188	0.110	−0.229
	*p*-value	0.884	0.137	0.389	0.068
L	Spearman correlation (Rho)	0.242	0.290	0.256	−0.207
	*p*-value	0.054	0.020	0.041	0.101
M	Spearman correlation (Rho)	0.093	0.034	0.137	−0.204
	*p*-value	0.464	0.788	0.281	0.107
N	Spearman correlation (Rho)	0.125	0.183	0.270	−0.354
	*p*-value	0.326	0.147	0.031	0.004
O	Spearman correlation (Rho)	0.162	0.323	0.318	−0.441
	*p*-value	0.202	0.009	0.010	<0.001
P	Spearman correlation (Rho)	0.486	0.460	0.507	−0.297
	*p*-value	<0.001	<0.001	<0.001	0.017
Q	Spearman correlation (Rho)	0.106	0.317	0.250	−0.299
	*p*-value	0.404	0.011	0.046	0.016
R	Spearman correlation (Rho)	0.304	0.370	0.434	−0.484
	*p*-value	0.015	0.003	<0.001	<0.001
S	Spearman correlation (Rho)	0.393	0.269	0.330	−0.332
	*p*-value	0.001	0.032	0.008	0.007
T	Spearman correlation (Rho)	0.253	0.274	0.333	−0.247
	*p*-value	0.044	0.028	0.007	0.049
U	Spearman correlation (Rho)	0.343	0.313	0.426	−0.321
	*p*-value	0.006	0.012	<0.001	0.010
V	Spearman correlation (Rho)	0.068	0.155	0.249	−0.303
	*p*-value	0.594	0.220	0.047	0.015
W	Spearman correlation (Rho)	0.205	0.192	0.191	−0.258
	*p*-value	0.105	0.128	0.130	0.039
X	Spearman correlation (Rho)	0.156	0.187	0.181	−0.141
	*p*-value	0.218	0.140	0.153	0.265
Y	Spearman correlation (Rho)	0.116	0.259	0.254	−0.449
	*p*-value	0.360	0.039	0.043	<0.001
Z	Spearman correlation (Rho)	0.425	0.506	0.506	−0.339
	*p*-value	<0.001	<0.001	<0.001	0.006

**Table 10:** Correlation of BIQLI with ER-SMFRS, blind SR-SMFRS, unblind SR-SMFRS, and SSSS.

		ER-SMFRS	Blind SR-SMFRS	Unblind SR-SMFRS	SSSS
1	Spearman correlation (Rho)	−0.330	−0.425	−0.433	0.509
	*p*-value	0.008	<0.001	<0.001	<0.001
2	Spearman correlation (Rho)	−0.369	−0.428	−0.451	0.544
	*p*-value	0.003	<0.001	<0.001	<0.001
3	Spearman correlation (Rho)	−0.319	−0.440	−0.398	0.536
	*p*-value	0.010	<0.001	0.001	<0.001
4	Spearman correlation (Rho)	−0.413	−0.473	−0.479	0.395
	*p*-value	<0.001	<0.001	<0.001	0.001
5	Spearman correlation (Rho)	−0.380	−0.516	−0.451	0.542
	*p*-value	0.002	<0.001	<0.001	<0.001
6	Spearman correlation (Rho)	−0.223	−0.336	−0.276	0.393
	*p*-value	0.076	0.007	0.027	0.001
7	Spearman correlation (Rho)	−0.172	−0.328	−0.265	0.235
	*p*-value	0.174	0.008	0.035	0.062
8	Spearman correlation (Rho)	−0.236	−0.309	−0.264	0.248
	*p*-value	0.061	0.013	0.035	0.048
9	Spearman correlation (Rho)	−0.149	−0.339	−0.303	0.409
	*p*-value	0.241	0.006	0.015	<0.001
10	Spearman correlation (Rho)	−0.199	−0.347	−0.334	0.477
	*p*-value	0.115	0.005	0.007	<0.001
11	Spearman correlation (Rho)	−0.468	−0.540	−0.533	0.454
	*p*-value	<0.001	<0.001	<0.001	<0.001
12	Spearman correlation (Rho)	−0.462	−0.532	−0.530	0.508
	*p*-value	<0.001	<0.001	<0.001	<0.001
13	Spearman correlation (Rho)	−0.361	−0.612	−0.554	0.388
	*p*-value	0.003	<0.001	<0.001	0.002
14	Spearman correlation (Rho)	−0.398	−0.572	−0.510	0.505
	*p*-value	0.001	<0.001	<0.001	<0.001
15	Spearman correlation (Rho)	−0.337	−0.550	−0.430	0.506
	*p*-value	0.006	<0.001	<0.001	<0.001
16	Spearman correlation (Rho)	−0.082	−0.327	−0.233	0.445
	*p*-value	0.518	0.008	0.064	<0.001
17	Spearman correlation (Rho)	−0.382	−0.458	−0.476	0.367
	*p*-value	0.002	<0.001	<0.001	0.003
18	Spearman correlation (Rho)	−0.273	−0.356	−0.331	0.384
	*p*-value	0.029	0.004	0.007	0.002
19	Spearman correlation (Rho)	−0.167	−0.281	−0.187	0.346
	*p*-value	0.188	0.025	0.140	0.005

## Data Availability

Not applicable.
